# Supplementary material for: Between Restrictive and Supportive Devices in the Context of Physical Restraints: Findings from a Large Mixed-Method Study Design
Source: Int J Environ Res Public Health. 2021 Dec 3;18(23):12764. doi: 10.3390/ijerph182312764 (PMC8657237; doi:10.3390/ijerph182312764)
Supplement: Supplementary file 1 [file ijerph-18-12764-s001.zip › ijerph-1444298-supplementary.pdf]

Table S1. Good Reporting of A Mixed-Methods Study (GRAMMS) [32].

Criteria	Page
(1) Describe the justification for using a mixed methods approach to the research question	3
(2) Describe the design in terms of the purpose, priority and sequence of methods	3
(3) Describe each method in terms of sampling, data collection and analysis	3-6
(4) Describe where integration has occurred, how it has occurred and who has participated in it	3 and 5-6
(5) Describe any limitation of one method associated with the present of the other method	13-14
(6) Describe any insights gained from mixing or integrating methods	6-14
